# Spatial Variability of Aroma Profiles of Cocoa Trees Obtained through Computer Vision and Machine Learning Modelling: A Cover Photography and High Spatial Remote Sensing Application

**DOI:** 10.3390/s19143054

**Published:** 2019-07-11

**Authors:** Sigfredo Fuentes, Gabriela Chacon, Damir D. Torrico, Andrea Zarate, Claudia Gonzalez Viejo

**Affiliations:** 1 School of Agriculture and Food, Faculty of Veterinary and Agricultural Sciences, University of Melbourne, Melbourne, VIC 3010, Australia; 2 Department of Wine, Food and Molecular Biosciences, Faculty of Agriculture and Life Sciences, Lincoln University, Lincoln 7647, New Zealand

**Keywords:** leaf area index, cocoa beans, volatile compounds, artificial neural networks, VitiCanopy app

## Abstract

Cocoa is an important commodity crop, not only to produce chocolate, one of the most complex products from the sensory perspective, but one that commonly grows in developing countries close to the tropics. This paper presents novel techniques applied using cover photography and a novel computer application (VitiCanopy) to assess the canopy architecture of cocoa trees in a commercial plantation in Queensland, Australia. From the cocoa trees monitored, pod samples were collected, fermented, dried, and ground to obtain the aroma profile per tree using gas chromatography. The canopy architecture data were used as inputs in an artificial neural network (ANN) algorithm, with the aroma profile, considering six main aromas, as targets. The ANN model rendered high accuracy (correlation coefficient (R) = 0.82; mean squared error (MSE) = 0.09) with no overfitting. The model was then applied to an aerial image of the whole cocoa field studied to produce canopy vigor, and aroma profile maps up to the tree-by-tree scale. The tool developed could significantly aid the canopy management practices in cocoa trees, which have a direct effect on cocoa quality.

## 1. Introduction

Cocoa (*Theobroma cacao* L) is considered a major world commodity crop and is the seventh most exported food product [1]. Its primary use is for chocolate manufacture, with an estimate of 3981 thousand tons of cocoa bean production in 2016 globally [2]. However, as with any other crop, the quality of cocoa beans is determinant for consumers’ acceptability and end-product cost. The cocoa beans intended for manufacturing must comply with specific quality parameters such as shape, size, color [3], flavor, aroma, cocoa butter, and protein content [4]. These are mainly related to the genotype of the clone, climatic and agricultural conditions, and agricultural practices (e.g., water management, fertilization, canopy management), as well as fermentation, drying, and industrialization processes [5,6,7,8,9]. The cocoa crop and its production are also very important for developing countries. For example, cocoa produce from Ecuador is highly valued internationally due to their recognized aroma intensity development [10].

Cocoa trees are grown in regions with high rainfall (1250–3000 mm/year), average temperatures between 18 and 32 °C, and calm wind conditions to avoid defoliation. Furthermore, shade is an important factor, especially in young trees, as it provides protection from excessive solar radiation and helps enhance aroma profiles that are desirable in the final chocolate product. They are usually planted at a density of 400–2500 trees per hectare and produce an average yield of between 200 and 800 kg of fermented dried cocoa per hectare [4,11,12].

There are three main cocoa tree varieties: (i) Criollo, (ii) Forastero, and (iii) Trinitario, the latter being a hybrid group. Varieties i and iii are considered as fine-quality or flavor-quality cocoa beans, while ii is used to produce bulk-quality cocoa. Specifically, the cocoa quality is mainly influenced by the growing conditions where the tree is grown, such as soil depth, pH (4.5–7), weather changes, and pest and disease control [13]. Some techniques to manage the cocoa trees to assure high-quality beans are: (i) Pest and disease control, (ii) soil nutrients management, (iii) weed control, and (iv) management of shade and canopy pruning [14]. The latter technique, (iv), is important due to the fact that the relationship between light and shade is critical, as the shade provides protection against sunlight and high winds, and lowers the temperature to manage the growth of the cocoa tree [4,11].

In cocoa plantations, some in-field factors that have been reported to affect cocoa quality are the exposure to solar radiation, which has effects on the final fruits’ dry weight and the growth temperature, which has, in turn, an influence on the lipid content and melting point [15]. However, no relationship between the aroma quality of the cocoa beans and canopy architecture parameters, such as leaf area index (LAI), has been reported. A complex method proposed by researchers to assess cocoa beans quality from the field is through the genetic identification of the variety by using deoxyribonucleic acid (DNA) fingerprinting and utilizing microsatellite markers containing the information from leaf samples [13,16]. However, this method is time-consuming, non-practical, and cost ineffective. Furthermore, the analysis of the trees at the leaf level has shown to be challenging due to the difficulty of sampling leaves with the same growth conditions (e.g., shade, age) [11] and the complexity of the environment when plants are grown between other tree species (i.e., within a forest).

This paper presents the assessment of canopy architecture parameters of cocoa trees using the VitiCanopy computer application [17] as a ground-truth to validate information extracted from aerial images, to automatically obtain these parameters within a plantation and up to a tree-by-tree resolution. From the same ground-truth cocoa trees, the volatile aroma compounds were assessed from fermented and dried cocoa beans by using gas chromatography–mass spectroscopy (GC–MS) with a solid phase microextraction in the headspace (SPME-HS). The canopy architecture data were used as inputs to develop a machine learning model to predict six of the main volatile compounds in cocoa beans and their associated aromas. Since all the information obtained either through computer vision algorithms (canopy architecture) and machine learning modeling (aroma profiles) was at the tree-by-tree scale, spatial distribution aroma maps were constructed from the cocoa plantation used in this research.

## 2. Materials and Methods

### 2.1. Site Description

The cocoa trees used in this research are located in a commercial farm with Papua New Guinea Hybrids (PNH) at Mt Edna Maria Creeks, (17°49′14″ S 146°02′21″ E), North Queensland, Australia. A map showing the location can be found as supplementary material in Figure S1. The soil type is clay loam with a pH of 5, under a tree sprinkler irrigation system with sprinkler discharge of 40 L per hour. Sprinklers were located between tree rows at 4 m intervals, and minimal chemical usage for pests and disease management was applied when required.

**Figure 1:**
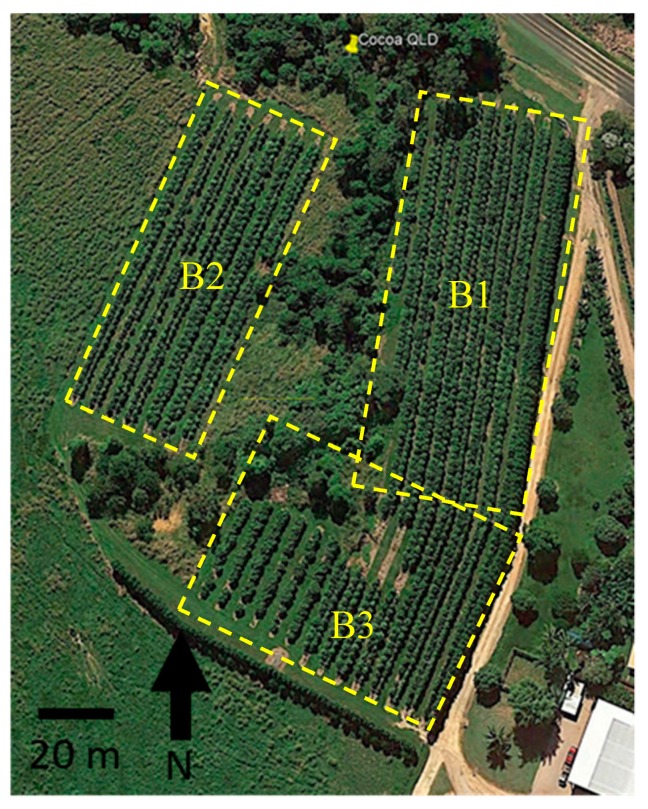
Aerial image of a cocoa plantation close to harvest from Mt Edna Maria Creeks, Queensland, Australia (Google Earth Pro™). Yellow dotted lines show the blocks used, numbered from 1 to 3.

Cocoa trees were planted in three blocks using a Tatura trellis pruning system, which requires a continuous shaping of the tree canopy. Measurements were only done in Blocks 1 and 2. Among Blocks 1 and 2, the planting density was 1000 trees ha^−1^ in a double-row layout, with distances of 5 m between rows and 2 m between trees. The blocks and rows were planted in different seasons for pruning test purposes. Block 1 was planted in March 2013, and Block 2 in October 2013. In both blocks, row A was planted first. The pattern area (Figure 1) was excluded from data collection because the trees were established as free-standing trees and were at the flowering stage. On the right side of Block 1 and at the lower lateral of the two blocks, the plantation is surrounded by *Erythrina variegata*, or coral tree, used as a windbreak, while Block 2 is surrounded by common vegetation of the site. The cocoa plantation was grown under full-sunlight conditions without intercropping species or shade species.

The weather data of the site were obtained at an approximate distance of 30 km from the plantation from the Bureau of Meteorology (BOM) weather station located at South Johnstone (Australian BOM Station No. 032037, 17.61° S 146.00° E). Average rainfall of 3289.3 mm per year, and mean values for maximum and minimum temperatures of 28.1 and 19.2 °C, respectively, were recorded in the season studied (2015–2016), which is consistent with the climate classification for the site as a tropical monsoonal region.

### 2.2. Cocoa Trees and Pods/Beans Sampling

A total of 24 cocoa trees were used for this study. Each tree was measured nine times to use as replicates. The cocoa pods were randomly sampled from the mentioned trees according to the general guidelines on sampling CAC/GL 50-2004, as recommended by the Codex Alimentarius Commission. Two to three cocoa pods were harvested from each tree. Each pod was assigned a label as B1 or B2 according to the blocks from which they were collected (one or two), followed by the number of the tree sampled (e.g., A15) and the letter of the row (A or B). Every tree was geo-located using the VitiCanopy App, which will be described following. The crops for the two blocks from this trial were harvested on the 2 February 2016.

### 2.3. Image Data Acquisition

**Figure 2:**
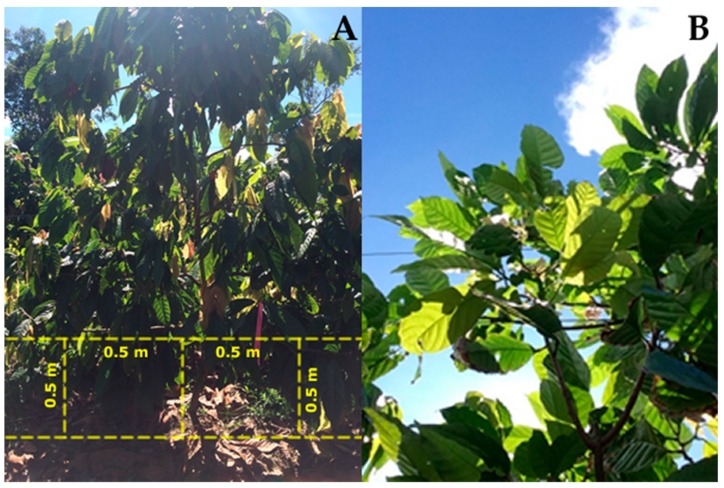
(**A**) Distance of image acquisition data in cocoa trees under a Tatura trellis system using the VitiCanopy application (app), and (**B**) image of canopy recorded using the VitiCanopy app.

For each of the 24 sampled trees, a total of nine upward-looking canopy images were obtained using the VitiCanopy computer application [17] within three consecutive days (three per day). The digital images from the tree canopies were obtained with the front camera of an iPhone 5S (Apple Inc. Corp. Cupertino, CA, USA), which has 1.2 megapixels of resolution. The device was mounted on a Soniq Bluetooth Selfie Stick (SONIQ Australia Pty Ltd., Braeside, Victoria, Australia) facing upwards at 0.5 m height using a tripod. The device was aligned using a bubble level attached to the selfie stick to ensure it was in a horizontal position for each image, obtained at a 0° Zenith angle. It was positioned with its maximum length perpendicular to the direction of the trunk. The images were acquired by locating the device at 0.5 m from the trunk and 0.5 m to the left and right sides (Figure 2). Imagery were obtained four hours before noon to avoid direct sunlight into the lens of the device, as recommended by Poblete-Echeverría et al. [18]. The dates of image acquisition were 29 January to 2 February 2016. All the equipment used for image acquisition were ubiquitous (smartphones), low-cost, and suitable for fieldwork.

### 2.4. Estimation of Canopy Architecture Parameters by the VitiCanopy App

Pictures with direct sunlight, weeds, posts, operators, and canopy from the other trees were considered unsuitable for analysis and discarded. Therefore, a total of 173 images were suitable for the analysis, with six to nine images analyzed from each tree. Canopy architecture parameters were obtained using the following algorithms to obtain the fractions of foliage projective cover (*f*_f_), crown cover (*f*_c_), and crown porosity (Φ), which were calculated from McFarlane et al. [19] as:

(1) $$f_{f}=1-\frac{t_{g}}{t_{p}}$$

(2) $$f_{c}=1-\frac{l_{g}}{t_{p}}$$

(3) $$\Phi =1-\frac{f_{f}}{f_{c}}$$

where *l*_g_ = large gap pixels; *t*_g_ = total pixels in all gaps, and *t*_p_ = total gap pixels.

LAI is calculated from Beer’s Law:

(4) $$LAI=f_{c}+\frac{\ln\Phi }{k}$$

where *k* is the coefficient of light extinction (*k* = 0.61) [20], and the clumping index at the zenith, Ω (0), was calculated as follows:

(5) $$\Omega \left(0\right)=\frac{\left(1-\Omega \right)\ln\left(1-f_{f}\right)}{\frac{\ln\left(\Phi \right)}{f_{f}}}$$

The clumping index is a correction factor to obtain the effective LAI (LAI_e_), which is the product of:

(6) $$LAI_{e}=LAI_{M}\times \Omega \left(0\right)$$

Equation (5) describes the non-random distribution of canopy elements. If Ω(0) = 1, that means that the canopy displays random dispersion. For Ω(0) > or < 1, the canopy is defined as clumped.

This innovative app estimates canopy architecture parameters using upward-looking digital images based on a method developed by Fuentes and De Bei et al. [21]. The image can be automatically sub-divided into *m* × *n* (*m* = number of rows, *n* = number of columns) sub-images for gap analysis. For this research, a sub-division of 5 × 5 was used for the VitiCanopy App and to analyze each tree sub-image from the aerial image analysis. The algorithms used in the VitiCanopy app were calculated based on the formulas from Macfarlane et al. [19]. In the settings menu of the application, the gap fraction threshold was set as 75%, which means that a big gap was considered when 75% of the pixels from the images corresponded to the sky [21]. A value of the light extinction coefficient (*k*) of 0.7 was set based on the range of the standard mean values identified from the samples and according to the literature review data of cocoa tree studies made by Yapp [22] and Daymond et al. [23]. Since in these images the sky serves as a background to isolate non-leaf material, the app filters sky and clouds automatically by segmentation of images using the blue channel of the RGB images. In order to record the Global Positioning System (GPS) data of each cocoa tree, the location service of the app was enabled. The images were analyzed with the application individually as they were obtained. The results from the app, which included the canopy architecture parameters, date, GPS location, and settings used were exported via email by tapping the “Export” option, which generates a comma separated values (.csv) file with all the data.

### 2.5. Aerial Imagery and Processing

**Figure 3:**
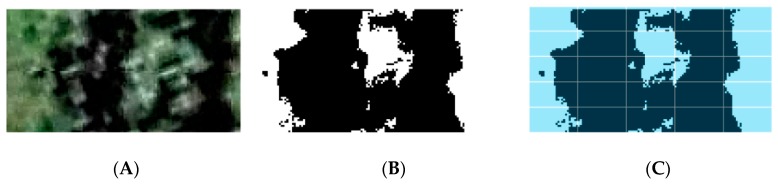
Analysis process from automatically cropped images per tree (**A**) and segmentation using the CieLab color code to obtain a binary image showing main tree biomass (**B**), and sub-division of 5 × 5 for gap analysis (**C**) and application of image analysis algorithms (Equations (1)–(6)).

The aerial image used for this research was obtained from Google Earth Pro (ver. 7.3.2.5776; Google, Mountain View, CA, USA), which uses a composite between satellite and aerial images. The only available image closer to the harvest was for 27 May 2016. However, it is expected from the biological point of view that the LAI will be similar within this month and that the trends between plants should also be maintained. The image was saved as a maximum high-resolution (4800 × 2679 pixels) Joint Photographic Expert Group (JPEG) file. The aerial image was processed to recognize every single plant and extract information from each one using the methodology proposed and described in previous studies [24,25] through customized codes written in Matlab^®^ (Mathworks Inc., Natick, MA, USA). In general, the automated procedure consists of the following steps:

1. Identification of the area designated by the plant (distance between plants and between rows). This was done by cropping the area for blocks and automatically sub-cropping each tree area for further analysis according to the distance between plants and the distance between rows [24,25] (Figure 3A).
2. Automatic Individual segmentation of tree biomass material using the CIELab (specifically the “a” band) color code to generate a binary image of sub-images analyzed per tree (Figure 3B).
3. Analysis of binary images per area assigned per plant using algorithms described previously (Equations (1)–(6)). Each sub-image is treated as an individual upward-looking image obtained by VitiCanopy, using the sky as a background. A subsequent subdivision of 5 × 5 was used for each tree image for gap analysis, as described by [17,21] (Figure 3C). From the aerial image, it was assumed to be a downward-looking image at Nadir (0° angle) using the inter-row as a background [17,21].
4. The obtained canopy architecture data were compiled in a matrix form to preserve plant positions and for easier handling for mapping purposes.
5. If canopies were of low cover (*f*_c_ > 0.1), they were considered as missing trees [24].
6. Tree GPS positions were extracted from the GPS coordinates of the corners of blocks for data mapping purposes.

### 2.6. Cocoa Bean Fermentation and Drying Process

The harvested cocoa pods were transported to The University of Melbourne, Australia, and stored for ten days at room temperature between 20 and 25 °C. The storage time allowed a significant increase of the pH related to cocoa flavor potential [26,27]. The seeds were taken from inside each pod sample and mixed with a solution of a commercial yeast *Saccharomyces cerevisiae* (Lowan Whole Foods, Glendenning, NSW, Australia) and water in a plastic container. For the fermentation process, the labeled plastic containers were placed into an incubator I100-G300-D (Thermoline Scientific Equipment Pty Ltd., Wetherill Park NSW, Australia), which was set up at 25 °C (0–12 h), 30 °C (12–24 h), 35 °C (24–36 h), 40 °C (36–48 h), 45 °C (48–72 h), and 48 °C (72–144 h). The temperature was monitored daily, and the seeds were manually flipped every 48 h to ensure aeration and uniform fermentation. After six days of fermentation, a cut test was performed to assess the quality of the process. Subsequently, for the drying process, the beans were set up in a Qualtex Solid State oven, Series 2000 (Qualtex Australasia P/L, Murarrie QLD, Australia) at 45 ± 2 °C, and mixed manually and daily for three days to get a uniform drying.

### 2.7. Analysis of Volatile Compounds Using Gas Chromatography-Mass Spectroscopy

The cocoa beans from each pod sample were ground in a commercial coffee blender Sunbeam Multigrinder II EM0405 (Sunbeam Products, Boca Raton, FL, USA) for 60 s. One gram of the ground beans was placed into a 15 mL tightly capped vial with 5 mL of saturated salt solution and 100 µL of pyrazine dilution. According to the procedure of De Brito et al. [5], the vials were mixed in a vortex (Vor-Mix VM80) for 1 min and preheated for 45 min at 60 °C, and then placed in a dry block heater and mixed in an orbital mixer OM1 (Ratek Instruments Pty. Ltd., Boronia, Victoria, Australia).

The volatile compounds were extracted by solid phase microextraction in the headspace (SPME-HS) with Supelco fibers 50/30 µm divinylbenzene/carboxene/polydimethylsiloxane (DVB/CAR/PDMS). The extraction of the volatile compounds of each sample took place for 15 min, with 30 min at 60 °C of fiber exposition to the cocoa aromas in the headspace [28]. The fibers were then analyzed by gas chromatography (GC; Agilent Technologies 6850 Series II, Network GC System) coupled to mass spectrometry (MS; Agilent Technologies 5973 Network Mass Selective Detector). The GC–MS was equipped with an Agilent J&W DB-Wax column (30 m × 0.25 mm, 0.25 µm film thickness). The oven temperature was set at 40 °C for 5 min, and then increased to 200 °C at a rate of 10 °C min^−1^, and finally maintained at 200 °C for 30 min. The carrier gas was helium (Air Liquide or BOC, Ultra-High Purity), with a linear velocity of 36 cm/s and a flow rate of 2.0 mL/min in constant flow mode. The SPME fiber was desorbed in the pulsed splitless injection mode for 5 min at 240 °C and opened after 0.5 min. The temperatures of the selective mass detector quadrupole were MS source 230 °C and MS Quad 180 °C, with an electronic impact ionization system at 70 eV. Compounds identification was performed by comparing the mass spectra of each compound with the National Institute of Standards and Technology MS NIST02.L (National Institute of Standards and Technology, Gaithersburg, MD, USA) library of mass spectra.

### 2.8. Statistical Analysis and Machine Learning Modeling

An analysis of variance (ANOVA) was conducted to assess significant differences in the LAI values and canopy architectures between the sampled trees, using the SAS^®^ 9.4 software (SAS Institute Inc., Cary, NC, USA). A post-hoc analysis with the Tukey studentized method (α = 0.05) was used. Means and standard deviation (SD) were calculated for each parameter.

**Figure 4:**
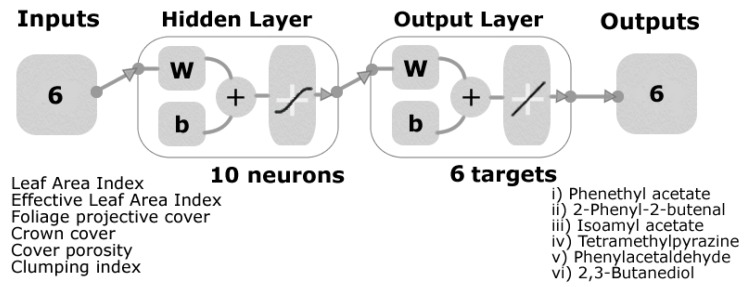
Model diagram showing the two-layer feedforward network with six inputs from the canopy architecture, 10 neurons in the hidden layer with a tan-sigmoid function, and a linear transfer function in the output layer. Six targets/outputs from the volatile compounds were used for the model.

A machine learning model was developed using the Bayesian Regularization training algorithm after testing 17 different artificial neural network (ANN) algorithms (two backpropagation using Jacobian derivatives, 11 backpropagation using gradient derivatives, and four supervised weight/bias training) using a customized and automatic Matlab^®^ R2018b developed code (unpublished). Six canopy architecture parameters from the canopy measurements ((i) LAI, (ii) LAI_e_, (iii) *f*_f_, (iv) *f*_c_, (v) ϕ, and (vi) Ω) were used as inputs to predict six main aroma compounds: (i) Phenethyl acetate, (ii) 2-phenyl-2-butenal, (iii) isoamyl acetate, (iv) tetramethylpyrazine, (v) phenylacetaldehyde, and (vi) 2,3-butanediol. All inputs and target values were normalized from −1 to 1. To develop the model, a random data division was used with 85% (N = 147) of the samples used for training, 15% (N = 26) for testing with a default derivative function, and a performance algorithm based on mean squared error (MSE). The model (Figure 4) was built with a two-layer feedforward network using a tan-sigmoid function in the hidden layer and a linear transfer function in the output layer. A trimming assessment was performed using 3, 5, 7, and 10 neurons to find the model with the best performance and less chance of overfitting. Statistical results are presented for the correlation coefficient (R) and mean squared error values (MSE).

Once developed, the machine learning model was run using the canopy architecture data extracted from the aerial imagery as inputs to obtain the six aromas per tree from the three blocks. Geo-location per tree was obtained from the GPS data from block corners and considering the plantation density through a customized code written in Matlab^®^ R2019a (Fuentes, unpublished). With this information, two maps of LAI_e_ and the aroma profiles were generated in Matlab, with the first using kriging interpolation algorithms and the second using scattered data representing each tree. A graphical representation of the complete methodology used in this study can be found in the supplementary material (Figure S2).

## 3. Results

### 3.1. Canopy Architecture and Volatile Compounds

Table 1 shows the results of the means and SD from the canopy architectures of each of the 24 sampled trees. There were significant differences (*p* < 0.05) between samples for the six parameters. However, for the clumping index, only one sample (B1C4A) was significantly different.

Table 2 shows the means and SD from the results of both blocks, in which significant differences (*p* < 0.05) can be observed for LAI, LAI_e_, and crown porosity (Φ). Block 1 had higher mean values of LAI and, although not significantly different, crown cover results compared to Block 2. On the contrary, Block 1 presented lower and significant values of clumping index than Block 2.

**Figure 5:**
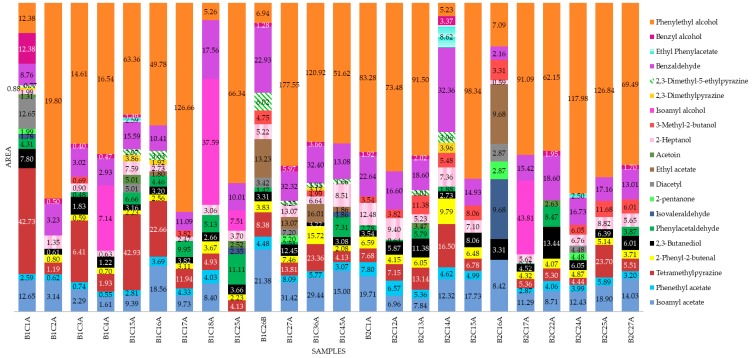
Peak areas of the volatile compounds found in the fermented cocoa bean samples. All values are reported in scientific notation 10^3^. Sample codes: B1 = Block 1, B2 = Block 2, C# = number of the tree, and A or B = position of tree (row).

Table 3 presents the volatile compounds found in the fermented cocoa beans and the aromas associated with them. It can be observed that most of the associated aromas are related to cocoa, butter, honey, fruity, floral, nutty, and roasted. Figure 5 shows the peak area of each volatile compound found in every sample. It can be seen that most of the samples had phenylethyl alcohol as the most abundant volatile compound, except for the beans of five trees (B1C1A, B1C18A, B1C26B, B2C14A, and B2C16A). Tetramethylpyrazine was present in most of the samples and is higher in those from Block 1 than Block 2. Benzaldehyde and 2,3-butanediol were present in all samples. However, the peak area varied between samples. Pehenthyl acetate and isoamyl acetate were present in all except for two (B1C25A and B2C16A) and one (B1C25A) sample(s), respectively. Other compounds, such as 2-pentanone (fruity) and ethyl acetate (pineapple/fruity/green) were only detected in four (B1C1A, B1C27A, B2C16A, and B2C24A) and six samples (B1C16A, B1C26B, B1C27A, B1C36A, B1C45A, and B2C16A), respectively.

### 3.2. Machine Learning Modeling

**Figure 6:**
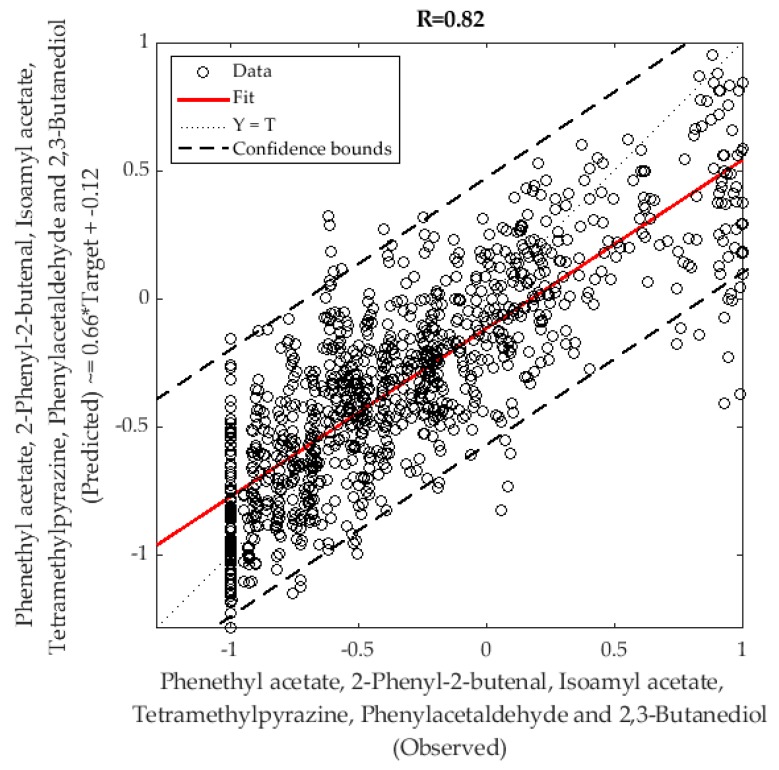
Artificial neural network overall model showing the observed responses (targets) in the x-axis and the predicted values (outputs) in the y-axis of the six volatile compounds. R = correlation coefficient.

Figure 6 shows the machine learning model to predict six of the main volatile compounds (Figure S3) in cacao beans (phenethyl acetate, 2-phenyl-2-butenal, isoamyl acetate, tetramethylpyrazine, phenylacetaldehyde, and 2,3-butanediol) using the LAI values and canopy architecture data as inputs. The overall model had 4.8% of outliers according to the 95% confidence bounds. In Table 4, it can be observed that the training stage had a correlation coefficient of R = 0.82, while the testing stage and the overall model had correlation coefficients of R = 0.81 and 0.82, respectively. Furthermore, the model had a high performance MSE = 0.09 for the training stage and MSE = 0.11 for the testing stage, which is indicative of an adequate model.

### 3.3. Implementation of the Machine Learning Model Developed to Map Aroma Compounds from Aerial Imagery

Aerial imagery allowed the extraction of canopy architecture parameters from sub-images per plant that are effectively comparable to upward-looking images from the VitiCanopy App. This was validated using a simple linear regression between the averaged LAI_e_ parameters from the 24 sampled trees in the field using VitiCanopy (observed) and those obtained from the same plants extracted from the aerial image (correlation coefficient (R) = 0.91; data not shown).

**Figure 7:**
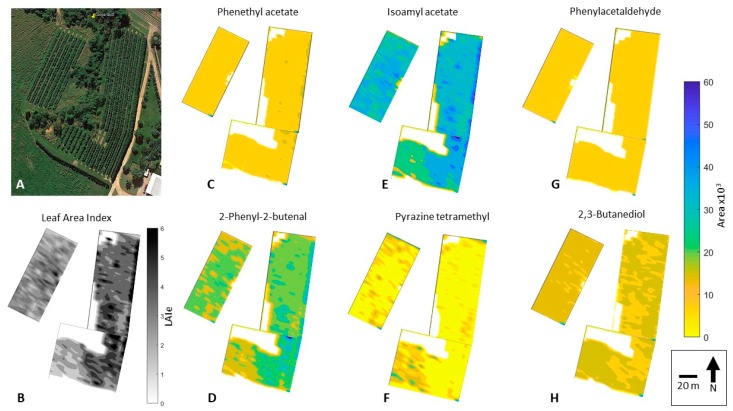
Original aerial image obtained from Google Maps Pro (**A**), and mapping of effective leaf area indexes (LAI_e_) (**B**) and aroma profiles for phenethyl acetate (honey/floral/yeasty/cocoa) (**C**); 2-phenyl-2-butenal (sweet/cocoa/nutty/beany) (**D**); isoamyl acetate (banana/fruity) (**E**); pyrazine tetramethyl (roasted/cocoa/coffee/nutty/earthy) (**F**); phenylacetaldehyde (honey/rose/cocoa) (**G**); and 2,3-butanediol (cocoa butter/creamy) (**H**).

**Figure 8:**
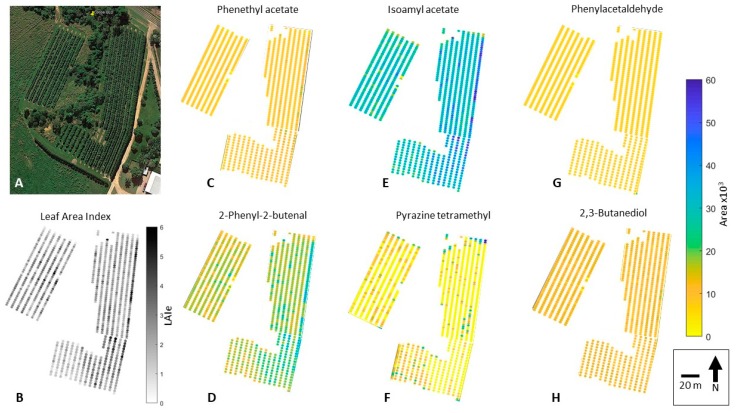
Original aerial image obtained from Google Map Pro (**A**), and plant-by-plant mapping of effective leaf area indexes (LAI_e_) (**B**) and aroma profiles for phenethyl acetate (honey/floral/yeasty/cocoa) (**C**); 2-phenyl-2-butenal (sweet/cocoa/nutty/beany) (**D**); isoamyl acetate (banana/fruity) (**E**); pyrazine tetramethyl (roasted/cocoa/coffee/nutty/earthy) (**F**); phenylacetaldehyde (honey/rose/cocoa) (**G**); and 2,3-butanediol (cocoa butter/creamy) (**H**).

The machine learning model developed and described in Figure 6 and Table 3 was implemented using the canopy architecture parameters obtained from the aerial imagery. Figure 7 shows the LAI_e_ and aroma profile data from each of the three blocks studied, using a kriging interpolation technique. The denormalized GC area values obtained from the machine learning model implementation were from 0 to 60 × 10^3^, which was used as a scale for all the aroma profiles. Since LAI_e_ is one of the most important canopy architectural parameters, it was individually mapped with values ranging from 0 (missing plants) to 6. Figure 8 shows the scattered values, which represent the LAI_e_ and aroma profile for individual plants from the field studied.

## 4. Discussion

### 4.1. Canopy Architecture and Volatile Compounds

The mean values of LAI and LAI_e_ for all trees fall within those reported by Daymond et al. [23] (2.2–4.5) for different clones of cacao in Brazil. The range of the mean values of canopy cover found in the present study was between 67% and 94%, which is similar to the range (72%–91%) obtained by Bos et al. [34] in Criollo and Forastero hybrids of cacao trees from Indonesia. The significant differences found between the two blocks for LAI, LAI_e_, and crown porosity may be due to the fact that Block 1 was planted in March 2013, while Block 2 was planted in October 2013, and therefore, their maturity stages were different at the time of measurements, giving an average LAI of 2.89 for Block 2, which is lower than that of Block 1 (3.35). High-resolution aerial images could be obtained using Unmanned Aerial Vehicle (UAV) platforms and high-resolution visible cameras. It is expected that by using the latter type of high-resolution images, the correlation between VitiCanopy and the aerial extraction of canopy architecture features will improve due to a more accurate gap analysis.

All volatile compounds found in the cocoa beans have been previously reported in other studies. Ten out of 20 of these compounds provide aromas related to nuts, roasted, cocoa, and chocolate, while six compounds provide fruity aromas including banana, cherry, and pineapple. Acetoin and 2,3-butanediol, which elicit creamy and butter aromas, were also found. According to Aprotosoaie et al. [35], the alcohols found in cocoa are due to fermentation and decrease with drying and roasting. However, 2-heptanol and phenylethyl alcohol, which give fruity and floral aromas, are commonly found in cacao. In the present study, phenylethyl alcohol was the most abundant compound in most of the samples. Furthermore, aldehydes and ketones, followed by esters and pyrazines, are the most important to determine cocoa quality [28,35], and in this study, seven compounds from aldehydes and ketones, four esters, and three pyrazines were found (Table 2). According to Rodriguez-Campos et al. [28], phenethyl acetate is an important ester in fermented cocoa beans, and higher concentrations of this compound are an indicator of high cocoa quality. In the present study, this volatile compound was identified in 22 out of 24 samples. On the other hand, similar to the present study, tetramethylpyrazine has been reported in other studies as the most abundant pyrazine and is one of the most important volatiles for cocoa flavor [36]. This was found in all except one sample from this study (Figure 5).

### 4.2. Machine Learning Modeling to Predict the Aroma Profile

Besides fermentation and drying, other factors that contribute to differences in aromas and flavors in cacao are the genotype, location and growth conditions, ripening, and amount of shade [37]. Therefore, the machine learning model developed in this study based on canopy architecture parameters has a strong basis on the physiological factors affecting the aroma profile. Hence, the high accuracy and performance obtained (R = 0.82; MSE = 0.09) using the canopy architecture information, which is related to the amount of shade, accurately represents the six volatile compounds commonly found in cocoa and that are determinant for its quality (Table 2). Up to date, there are no known studies that have managed to predict the volatile compounds in cacao using the canopy architecture. However, there was a study in grapevines in which flavonol contents were predicted using crown porosity and LAI using partial least squares (PLS) regression [38].

The model presented in this paper may be useful by producers to modify the canopy architecture of the cocoa trees according to the desired aroma characteristics on the final product. This would allow them to avoid financial loses or predict the end use of the final product by anticipating the quality based on the aroma profile. This model may be fed with more data from samples of other regions and genotypes to create a general predictive model to be used by any grower. On the other hand, similar models may be developed for other crops to predict and improve the quality at early stages.

### 4.3. Implementation of the Machine Learning Model Developed to Map Aroma Compounds from Aerial Imagery

Spatial distribution of LAI_e_ and aroma profiles were possible due to high-resolution aerial images, which can be effectively used to implement the canopy cover photography algorithms described (Equations (1)–(6)). A similar approach was implemented for grapevines using *f*_c_, with similar results from imagery obtained using unmanned aerial vehicles [24]. From the kriging interpolated maps (Figure 7) it can be seen that some compounds are highly dependent on vigor characteristics of plants, such as isoamyl acetate, with clear differences of higher areas found for the far-right row in Block 1 (Figure 7E). These trees corresponded to almost double LAI_e_ values (~5) compared to the average of trees in Blocks 1 and 2 (Figure 7A,B). Other compounds, such as phenethyl acetate, phenylacetaldehyde, and pyrazine tetramethyl, do not have many spatial differences among the three blocks (Figure 7C,G,F, respectively). The latter results confirm that the pattern of the machine learning models was effectively identified, and they are independent for each aroma related to canopy architecture parameters.

The differences in the spatial distribution of growth and aroma profiles can be more clearly seen using a scattering plot, which represents the tree-by-tree scale (Figure 8). This spatial distribution map could offer growers a powerful tool to perform tailored canopy management strategies to manage aroma profile outputs that could be considered desirable or to obtain a uniform quality of pods and, finally, the chocolate produced.

## 5. Conclusions

The data analysis tools presented in this paper could contribute significantly to adapting management strategies from growers, specifically in relation to canopy management, to obtain desired aroma profiles at the tree-by-tree scale. Specific practical applications could be related to homogenize the aroma quality profile within plants and the entire plantation or to increase desired and specific aromas to change the style of the cocoa volatiles. These management strategies can be implemented within the critical stages of the growing period of cocoa trees, or close to harvest. By implementing VitiCanopy and the machine learning modeling strategies presented, these potential changes in the volatile composition of beans related to canopy architecture changes due to canopy management can be obtained in real time at the fingertips of growers through their portable electronic devices (smartphones and tablet PCs). The latter will enhance crop management capabilities from major industries, but more importantly, by growers in developing countries.

## Figures and Tables

**Table 1:** Means and standard deviation (SD) of the parameters obtained from the canopy architecture for each sampled tree. Different letters (superscripts) represent statistically significant differences between samples using the Tukey studentized test with α = 0.05.

Sample	LAI		LAI_e_		Canopy Cover (*f*_c_)		Crown Cover (*f*_f_)		Crown Porosity (Φ)		Clumping Index (Ω)	
	Mean	SD	Mean	SD	Mean	SD	Mean	SD	Mean	SD	Mean	SD
B1C15A	3.08 ^b^	0.68	2.79 ^a^	0.76	0.836 ^a^	0.094	0.962 ^a^	0.061	0.134 ^a^	0.050	0.951 ^a^	0.067
B1C16A	2.78 ^b^	0.96	2.55 ^a^	1.12	0.755 ^a^	0.116	0.879 ^a^	0.107	0.142 ^a^	0.050	0.851 ^a^	0.131
B1C17A	3.60 ^a^	0.69	3.19 ^a^	0.36	0.868 ^a^	0.063	0.957 ^a^	0.060	0.094 ^b^	0.035	0.904 ^a^	0.122
B1C18A	3.47 ^a^	0.82	3.18 ^a^	1.08	0.843 ^a^	0.094	0.932 ^a^	0.077	0.097 ^b^	0.036	0.887 ^a^	0.127
B1C1A	3.62 ^a^	0.68	3.32 ^a^	0.98	0.863 ^a^	0.069	0.945 ^a^	0.050	0.088 ^b^	0.032	0.888 ^a^	0.100
B1C25A	4.56 ^a^	0.73	4.08 ^a^	0.73	0.939 ^a^	0.037	0.994 ^a^	0.014	0.056 ^b^	0.026	0.986 ^a^	0.034
B1C26B	2.55 ^b^	0.47	2.33 ^a^	0.69	0.730 ^a^	0.074	0.863 ^a^	0.086	0.153 ^a^	0.050	0.814 ^a^	0.112
B1C27A	3.31 ^a^	0.70	3.04 ^a^	0.97	0.834 ^a^	0.105	0.928 ^a^	0.087	0.104 ^b^	0.030	0.894 ^a^	0.121
B1C2A	3.50 ^a^	1.16	3.20 ^a^	1.33	0.827 ^a^	0.129	0.915 ^a^	0.111	0.099 ^b^	0.051	0.875 ^a^	0.146
B1C36A	3.68 ^a^	0.20	3.32 ^a^	0.56	0.867 ^a^	0.046	0.946 ^a^	0.054	0.080 ^b^	0.011	0.872 ^a^	0.117
B1C3A	4.29 ^a^	1.20	3.92 ^a^	1.43	0.881 ^a^	0.139	0.935 ^a^	0.141	0.059 ^b^	0.031	0.905 ^a^	0.195
B1C45A	2.65 ^b^	0.45	2.41 ^a^	0.67	0.772 ^a^	0.079	0.913 ^a^	0.070	0.156 ^a^	0.030	0.886 ^a^	0.092
B1C4A	2.40 ^b^	0.29	2.18 ^b^	0.51	0.668 ^b^	0.085	0.775 ^b^	0.112	0.135 ^a^	0.036	0.723 ^b^	0.116
B2C12A	3.06 ^b^	0.48	2.84 ^a^	0.75	0.847 ^a^	0.061	0.980 ^a^	0.041	0.136 ^a^	0.035	0.972 ^a^	0.056
B2C13A	3.33 ^a^	0.49	3.03 ^a^	0.56	0.875 ^a^	0.046	0.991 ^a^	0.017	0.117 ^b^	0.035	0.986 ^a^	0.028
B2C14A	2.61 ^b^	0.98	2.46 ^b^	1.13	0.727 ^b^	0.196	0.853 ^a^	0.209	0.152 ^a^	0.053	0.863 ^a^	0.158
B2C15A	2.30 ^b^	0.29	2.12 ^b^	0.48	0.720 ^b^	0.059	0.888 ^a^	0.068	0.188 ^a^	0.035	0.864 ^a^	0.073
B2C16A	2.81 ^b^	0.51	2.59 ^a^	0.66	0.791 ^a^	0.114	0.919 ^a^	0.122	0.140 ^a^	0.021	0.902 ^a^	0.132
B2C17A	2.88 ^b^	0.89	2.63 ^a^	0.89	0.770 ^a^	0.145	0.885 ^a^	0.126	0.134 ^a^	0.053	0.871 ^a^	0.139
B2C1A	3.27 ^a^	0.49	2.99 ^a^	0.55	0.867 ^a^	0.048	0.984 ^a^	0.031	0.119 ^b^	0.028	0.974 ^a^	0.052
B2C22A	2.91 ^b^	0.36	2.69 ^a^	0.60	0.816 ^a^	0.059	0.947 ^a^	0.058	0.138 ^a^	0.027	0.913 ^a^	0.085
B2C24A	2.56 ^b^	0.76	2.36 ^b^	0.81	0.719 ^b^	0.150	0.849 ^a^	0.159	0.153 ^a^	0.063	0.828 ^a^	0.154
B2C25A	3.02 ^b^	0.63	2.80 ^a^	0.83	0.796 ^a^	0.120	0.901 ^a^	0.122	0.118 ^b^	0.030	0.865 ^a^	0.140
B2C27A	3.13 ^a^	0.64	3.13 ^a^	0.64	0.849 ^a^	0.060	0.976 ^a^	0.036	0.131 ^a^	0.038	0.962 ^a^	0.055

Abbreviations: LAI = Leaf Area Index, LAI_e_ = effective LAI; sample codes: B1 = Block 1, B2 = Block 2, C# = number of the tree, and A or B = position of the tree (row). Means were taken from six to nine images per tree.

**Table 2:** Means and standard deviation (SD) of canopy architecture parameters for the two blocks. Different letters represent statistically significant differences between samples using the Tukey studentized test with α = 0.05.

Block	LAI		LAI_e_		Canopy Cover (*f*_c_)		Crown Cover (*f*_f_)		Crown Porosity (Φ)		Clumping Index (Ω)	
	Mean	SD	Mean	SD	Mean	SD	Mean	SD	Mean	SD	Mean	SD
B1	3.35 ^a^	0.93	3.04 ^a^	1.01	0.822 ^a^	0.11	0.919 ^a^	0.095	0.107 ^b^	0.048	0.880 ^a^	0.126
B2	2.89 ^b^	0.67	2.68 ^b^	0.76	0.796 ^a^	0.12	0.923 ^a^	0.116	0.139 ^a^	0.043	0.907 ^a^	0.116

Abbreviations: LAI = Leaf Area Index, LAI_e_ = effective LAI, B1 = Block 1, B2 = Block 2.

**Table 3:** Major volatile compounds found in the fermented cocoa beans, and the associated aromas.

Volatile Compounds	Compound Group	Aroma Associated	References
*Phenethyl acetate*	*Ester*	*Honey/floral/yeasty/cocoa*	[28,29]
*2-Phenyl-2-butenal*	*Aldehyde*	*Sweet/cocoa/nutty/beany*	[28,29]
*Isoamyl acetate*	*Ester*	*Banana/fruity*	[29,30]
*Tetramethylpyrazine*	*Pyrazine*	*Roasted/cocoa/coffee/nutty/earthy*	[7,29,31]
*Phenylacetaldehyde*	*Aldehyde*	*Honey/rose/cocoa*	[29,31]
*2,3-Butanediol*	*Alcohol*	*Cocoa butter/creamy*	[29,30]
Isovaleraldehyde	Aldehyde	Chocolate/nutty/cocoa	[28,29]
2-Pentanone	Ketone	Fruity	[29,32]
Diacetyl/2,3-Butanedione	Ketone	Butter/creamy/caramel	[28,29]
Ethyl acetate	Ester	Pineapple/fruity/green	[28,29]
Acetoin	Ketone	Butter/creamy/dairy/fatty	[28,29]
2-Heptanol	Alcohol	Citrus/fruity	[28,29]
3-Methyl-2-butanol	Alcohol	Cocoa/alcoholic/musty	[29]
Isoamyl alcohol	Alcohol	Malty/banana/pungent	[29,32]
2,3-Dimethylpyrazine	Pyrazine	Caramel/cocoa/coffee/nut skin	[29,33]
2,3-Dimethyl-5-ethylpyrazine	Pyrazine	Burnt/roasted/cocoa	[29]
Benzaldehyde	Aldehyde	Cherry/almond/bitter	[29,33]
Ethyl Phenylacetate	Esters	Honey/rose/dark chocolate	[29]
Benzyl alcohol	Alcohol	Sweet/floral/rose/phenolic	[28]
Phenylethyl alcohol	Alcohol	Honey/bready/floral	[29]

Compounds in italics were used as targets for machine learning modeling.

**Table 4:** Results from the artificial neural network model showing the numbers of samples and observations, correlation coefficient (R), and performance based on mean squared error (MSE) for each stage.

Stage	Samples	Observations (Samples × 6 Targets)	R	MSE
Training	147	882	0.82	0.09
Testing	26	156	0.81	0.11
Overall	173	1038	0.82	-

## Supplementary Materials

The following are available online at https://www.mdpi.com/1424-8220/19/14/3054/s1, Figure S1. Map showing the site location where the study was conducted. The image was obtained from Google Earth (Google Technology company, Menlo Park, CA, USA); Figure S2. Diagram representing the methods used in the study, where (A) represents the canopy architecture assessment at ground level using the VitiCanopy app, (B) depicts the cacao pods processing and assessment of volatile compounds using gas chromatography, mass spectroscopy, (C) the development of the machine learning modelling using (A) as inputs and (B) as targets/outputs, (D) represents the canopy architecture assessment from aerial images using a customized Matlab^®^ code, and (E) the development of spatial distribution maps using the outputs from (C) using the data from (D) as inputs; Figure S3. Peak areas of the six volatile compounds found in the fermented cocoa bean samples used to develop the machine learning model. All values are reported in scientific notation 10^3^. Sample codes: B1 = Block 1, B2 = Block 2, C# = number of the tree, and A or B = position of the tree (row).
