# Supplementary material for: Spatial Variability of Aroma Profiles of Cocoa Trees Obtained through Computer Vision and Machine Learning Modelling: A Cover Photography and High Spatial Remote Sensing Application
Source: Sensors (Basel). 2019 Jul 11;19(14):3054. doi: 10.3390/s19143054 (PMC6678375; doi:10.3390/s19143054)
Supplement: Supplementary file 1 [file sensors-19-03054-s001.pdf]

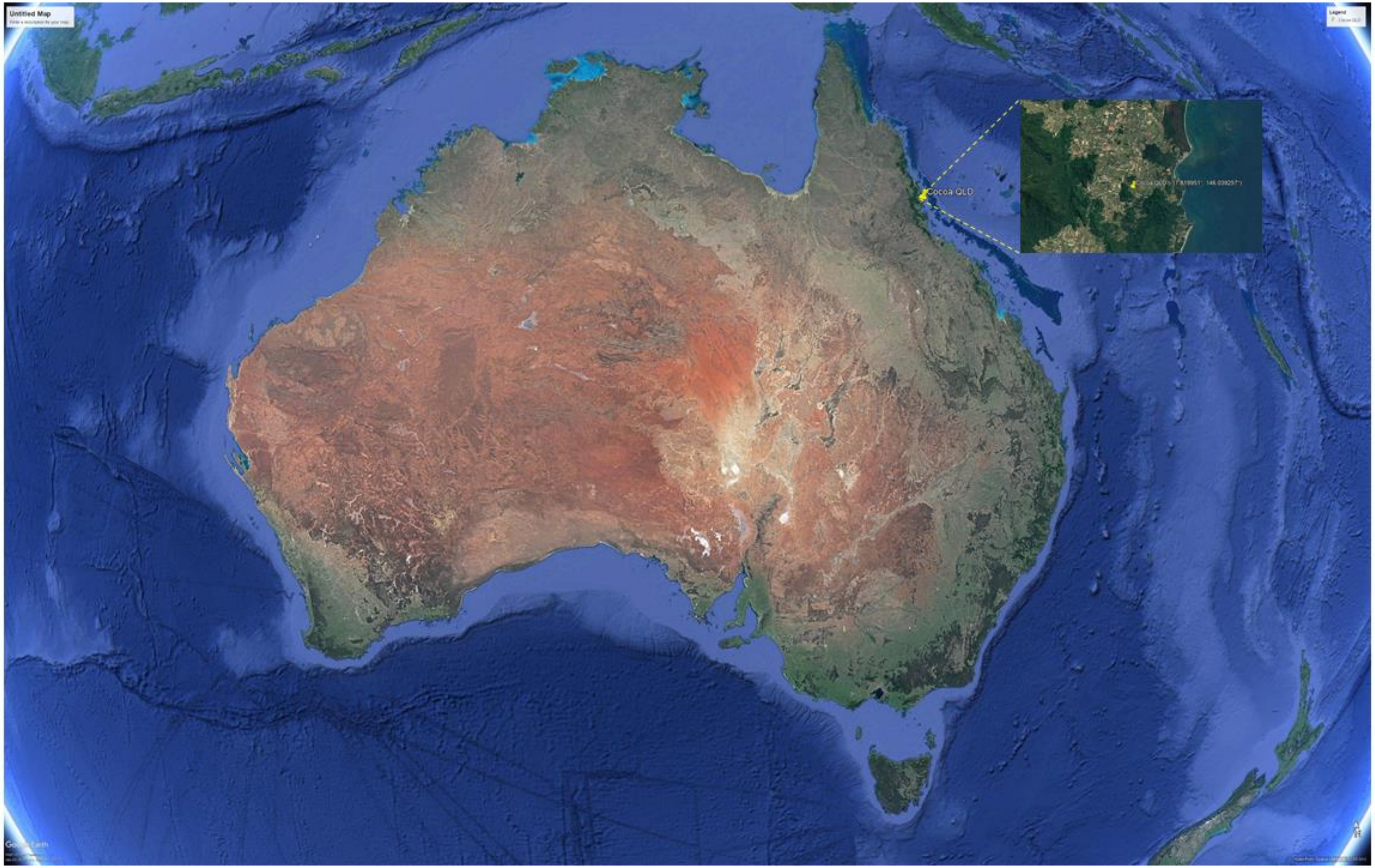

Figure S1. Map showing the site location where the study was conducted. Image was obtained from Google Earth (Google Technology Company, Menlo Park, CA, USA)

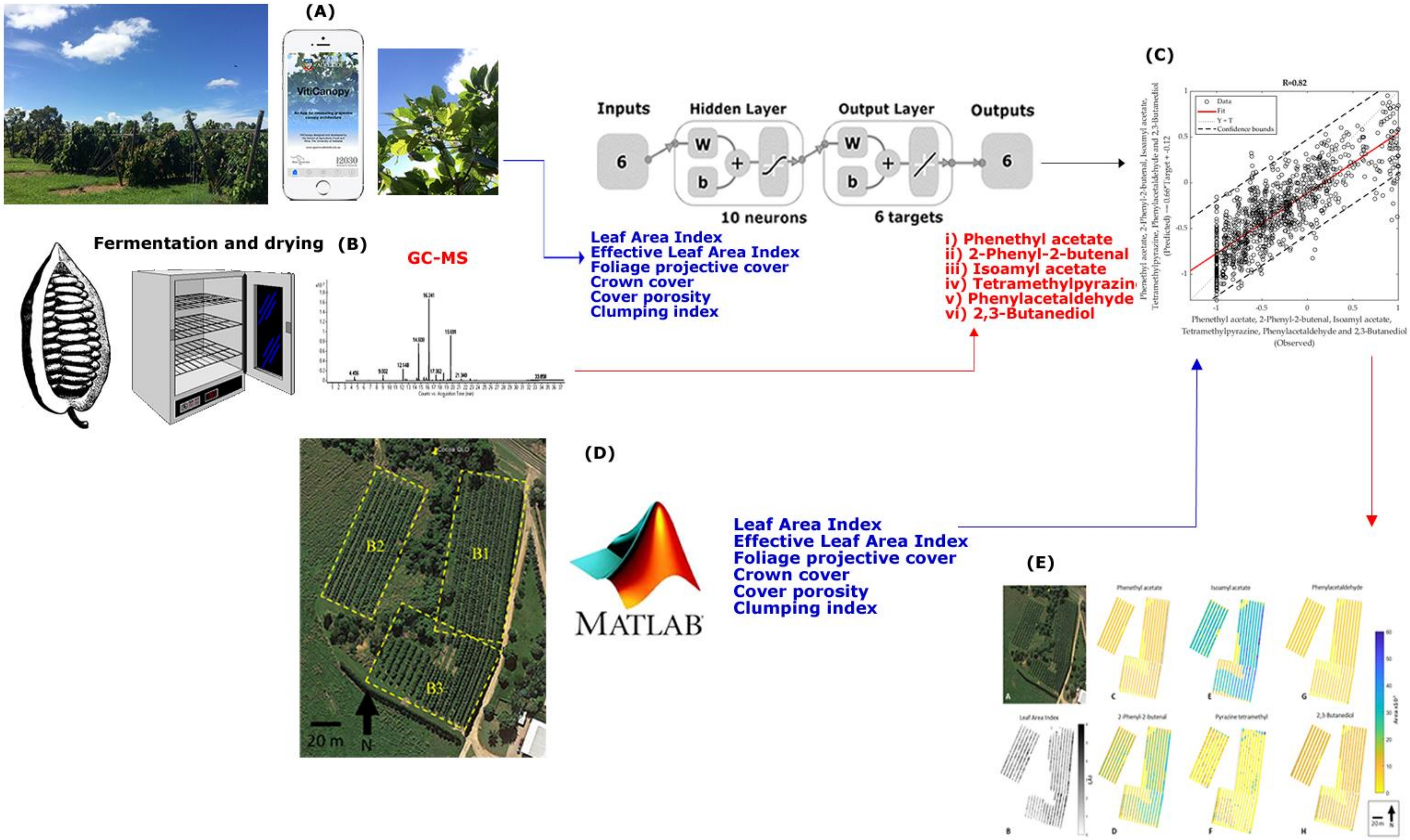

Figure S2. Diagram representing the methods used in the study, where (**A**) represents the canopy architecture assessment at ground level using the VitiCanopy app, (**B**) depicts the cacao pods processing and assessment of volatile compounds using gas chromatography, mass spectroscopy, (**C**) the development of the machine learning modelling using (A) as inputs and (B) as targets/outputs, (**D**) represents the canopy architecture assessment from aerial images using a customized Matlab® code, and (**E**) the development of spatial distribution maps using the outputs from (**C**) using the data from (**D**) as inputs.

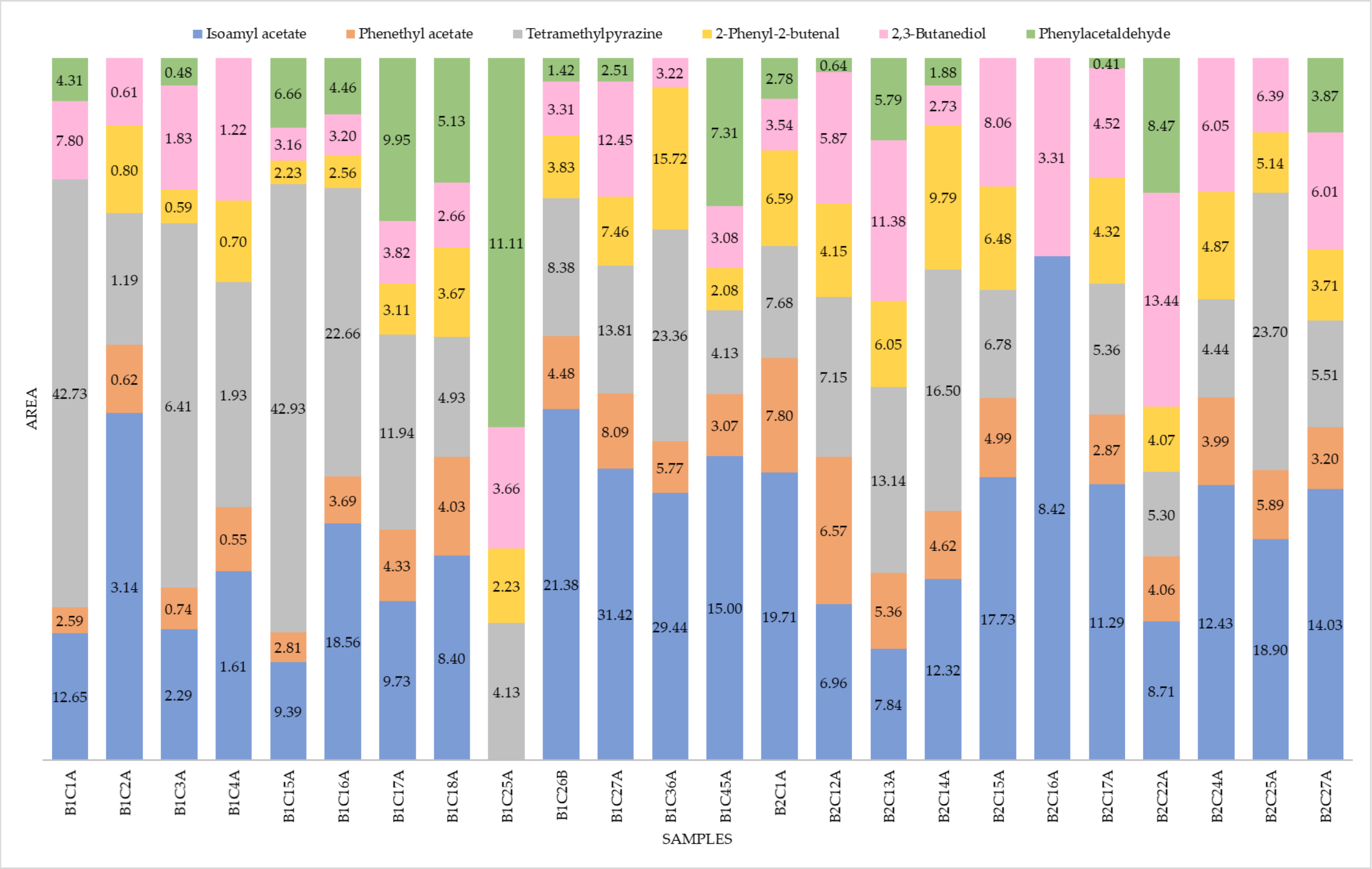

Figure S3. Peak areas of the six volatile compounds found in the fermented cocoa bean samples used to develop the machine learning model. All values are reported in scientific notation 10^3. Sample codes: B1 = Block 1, B2 = Block 2 0.25 cm, C# = number of the tree, and A or B = position of the tree (row).
